# Advancing the application of systems thinking in health: realist evaluation of the Leadership Development Programme for district manager decision-making in Ghana

**DOI:** 10.1186/1478-4505-12-29

**Published:** 2014-06-16

**Authors:** Aku Kwamie, Han van Dijk, Irene Akua Agyepong

**Affiliations:** 1 University of Ghana, School of Public Health, P.O. Box LG 13, Accra, Ghana; 2 Wageningen University, Anthropology and Sociology of Development, P.O. Box 8130, Hollandseweg 1 6700, EW, The Netherlands

**Keywords:** Continuous quality improvements, District health systems, Realist evaluation, Systems thinking

## Abstract

**Background:**

Although there is widespread agreement that strong district manager decision-making improves health systems, understanding about how the design and implementation of capacity-strengthening interventions work is limited. The Ghana Health Service has adopted the Leadership Development Programme (LDP) as one intervention to support the development of management and leadership within district teams. This paper seeks to address how and why the LDP ‘works’ when it is introduced into a district health system in Ghana, and whether or not it supports systems thinking in district teams.

**Methods:**

We undertook a realist evaluation to investigate the outcomes, contexts, and mechanisms of the intervention. Building on two working hypotheses developed from our earlier work, we developed an explanatory case study of one rural district in the Greater Accra Region of Ghana. Data collection included participant observation, document review, and semi-structured interviews with district managers prior to, during, and after the intervention. Working backwards from an in-depth analysis of the context and observed short- and medium-term outcomes, we drew a causal loop diagram to explain interactions between contexts, outcomes, and mechanisms.

**Results:**

The LDP was a valuable experience for district managers and teams were able to attain short-term outcomes because the novel approach supported teamwork, initiative-building, and improved prioritisation. However, the LDP was not institutionalised in district teams and did not lead to increased systems thinking. This was related to the context of high uncertainty within the district, and hierarchical authority of the system, which triggered the LDP’s underlying goal of organisational control.

**Conclusions:**

Consideration of organisational context is important when trying to sustain complex interventions, as it seems to influence the gap between short- and medium-term outcomes. More explicit focus on systems thinking principles that enable district managers to better cope with their contexts may strengthen the institutionalisation of the LDP in the future.

## Background

To date, the majority of management and leadership initiatives in low- and middle-income countries (LMICs) have focused on skills acquisition [1], with less attention paid to the complexity of the contexts and the health system arrangements which support or hinder such initiatives. In this paper we explore, using realist evaluation methodology, the outcomes, contexts, and mechanisms of a management and leadership initiative introduced into the district health system in the Greater Accra Region of Ghana, and whether or not such an intervention supports systems thinking in district managers. Firstly, we present an extensive background of the decision-making context at district-level. We then delve into several concepts, including systems thinking and continuous quality improvements, before discussing our analytical framework, case study design, results, and conclusions.

### District manager decision-making and systems thinking

In district health systems in LMICs, district managers link the national and regional levels – where policies are formulated – to the facility and community levels – where services are delivered. District managers are responsible for providing management and leadership to supervise staff, balance resources, coordinate programmes, and network with local officials and community members, all in a specific time and place. Thus, the manner in which district managers make decisions is important. It has been argued that limited management and leadership capacities at district level contribute to bottlenecks in achieving health outcomes [2-5].

Questions pertaining to management and leadership are some of the most complex in health systems analyses, not least because developing management and leadership requires nurturing myriad individual and organisational capacities [6]. Consequently, interventions that aim to strengthen management and leadership are also complex, and engage with both individual and organisational processes. District managers find themselves navigating complex environments in which district health systems display features of complex adaptive systems, such as self-organisation, path-dependence, emergence, and feedback loops. District health systems evolve over time as a result of multiple interactions between individuals and the system’s structure [7,8]. As an approach to navigating this complexity, systems thinking aims to identify the interrelations between a system’s various components [9]. Defined by de Savigny and Adam, “s*ystems thinking is an approach to problem*-*solving that views* ‘*problems*’ *as part of a wider*, *dynamic system*” [10]. The authors further identify a cluster of problem-solving skills relevant for systems thinking that distinguishes it from ‘usual thinking’ paradigms (Table 1). Due to their vantage point at the helm of district health systems, systems thinking can usefully support district manager decision-making.

**Table 1:** Systems thinking skills

**From ‘usual thinking’ approaches…**	**…to systems thinking**
Focused on particular events (*Static thinking*)	Problems framed in terms of a patterns of behaviour over time (*Dynamic thinking*)
Focused on particular details (*Tree*-*by*-*tree thinking*)	Focused on understanding the context of relationships (*Forest thinking*)
Focused on factors that influence/correlate with results (*Factors thinking*)	Focused on causality and understanding how behaviour is generated (*Operational thinking*)
System-generated behaviours are driven by external forces (*Systems*-*as*-*effect thinking*)	System-generated behaviours are driven by internal actors who interact with system itself (*Systems*-*as*-*cause thinking*)
Causality is viewed as uni-directional, without interdependence or interactions between causes (*Straight*-*line thinking*)	Causality is viewed as ongoing with feedback effects, including interdependence and interactions between causes (*Loop thinking*)

(*Adapted from Richmond*, *2000*[11]).

### Continuous quality improvements

Continuous quality improvement (CQI) is both a management philosophy and approach. Adopted in American healthcare institutions in the 1980s, the concept spread to LMICs during the 1990s. CQI offers a systematic way of supporting change in management processes towards improving the organisational culture of quality [12]. CQI is based on the assumption that problems within organisations are not rooted clinically or administratively, but are rather systemic and arise out of structural inabilities to perform as intended [13]. McLaughlin and Kaluzny identify nine elements necessary to classify an approach as CQI (Table 2). Systems thinking is embedded within this constellation, and can be seen as the glue that binds CQI elements together (the authors refer to this as ‘systems-view’).

**Table 2:** Elements of continuous quality improvements

**Element**	**Description**
Systems-view	Emphasis on analysis of the whole system providing a service, or influencing an outcome
Customer focus	Emphasis on both customer (patient, provider, payer) satisfaction and health outcomes as performance measures
Data-driven analysis	Emphasis on gathering and use of objective data on system operations and system performance
Implementer involvement	Emphasis on involving the owners of all components of the system in seeking a common understanding of its delivery process
Multiple causation	Emphasis on identifying the multiple root causes of a set of system phenomena
Solution identification	Emphasis on seeking a set of solutions that enhance overall system performance though simultaneous improvements in a number of normally independent functions
Process optimisation	Emphasis on optimising a delivery process to meet customer needs regardless of existing precedents, and on implementing the system changes regardless of existing territories and fiefdoms
Continuing improvement	Emphasis on continuing the systems analysis, even when a satisfactory solution to the presenting problem is obtained
Organisational learning	Emphasis on organisational learning so that the capacity of the organisation to generate process improvement and foster personal growth is enhanced

(*Adapted from McLaughlin and Kaluzny*, *1994*[13]).

To date, the impact of CQI in sub-Saharan Africa has been mixed. Case studies from three countries have demonstrated several factors that contribute to reduced CQI sustainability and effectiveness [14]. These are: i) introducing quality management as a vertical programme; ii) lacking systemic perspectives and identifying problems in their own sub-systems; iii) oversimplifying decision-making through the use of toolbox techniques; and iv) the conundrum of organisational culture and quality management: does organisational culture change to modify practise, or does organisational culture change by modifying practise? Furthermore, CQI is always implemented within an organisation’s own context – its history, cultural norms, and values. This latter point contributes to understanding the ‘inherent duality’ of CQI, namely that its principles are based on two distinct, paradoxical goals: although CQI promotes organisational control, uniformity, and standardisation, it also gives rise to organisational creativity, learning, and cultural change. This means that CQI practise (and the mechanisms behind it) will vary depending on whether its underlying goal is organisational control or organisational learning. Related to this, CQI’s underlying goal will be driven, either implicitly or explicitly, by the culture and structure of the organisation itself. Sitkin et al. [15], suggest that the most likely goal is informed by the degree of organisational uncertainty: when uncertainty is high, the organisation is predisposed to learning because control, in a sense, is out of reach. On the other hand, when contextual uncertainty is low, the organisation is predisposed to control because the problem is well understood and can be dealt with mechanistically. CQI has been proposed as a potential solution to improving service delivery in Ghana [16].

### Implementing the Leadership Development Programme in the Greater Accra Region

In Ghana, district managers are staff of the Ghana Health Service (GHS)^a^. The context of district manager decision-making is such that resource decisions (human, material, and financial) are constrained. This is partly due to the hierarchical structure of the GHS in which decision-making remains highly centralised, and resources scarce [17,18]. District managers have more discretion around programming decisions. Formalised management training is limited, and most managers learn their management roles on the job. Additionally, managers face serious time constraints due to concurrent scheduling of vertical and donor programme activities.

**Figure 1:**
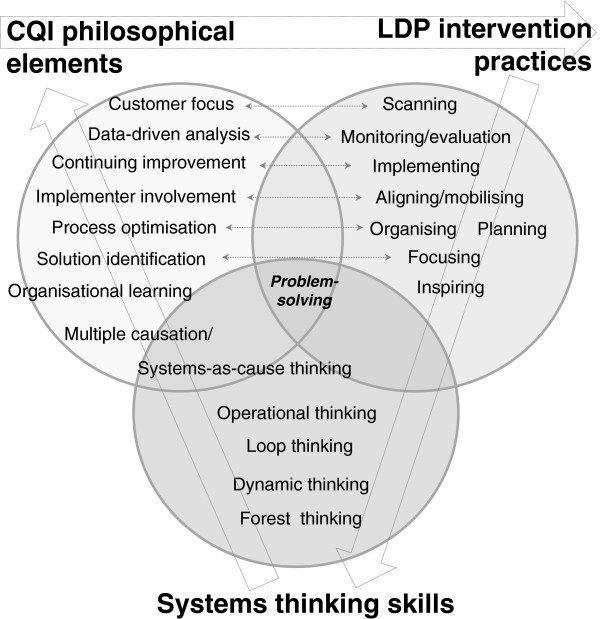
**Overlap in problem**-**solving approach between systems thinking**, **CQI, and the LDP.**

The LDP has been intermittently implemented in Ghana since 2008. Developed by Management Sciences for Health [19], the LDP has been implemented in several countries including Egypt [20], Kenya [21,22], and Mozambique [23]; the Greater Accra Region first introduced the LDP in 2010. In 2011, the LDP was proposed as an approach to address limited responsiveness, lacking leadership, and mismatched resources indicated as bottlenecks to improving maternal and newborn (MNH) service delivery [24]^b^. The LDP is designed for teams to apply ‘leading and managing’ practices to service delivery problems (referred to as ‘challenges’ in the LDP – Table 3). This is realised through teamwork, defining root causes, action planning, monitoring, and evaluation, and repeating the cycle. Its programme theory puts forth that, when deployed in tandem, leading and managing practices improve work climate, management systems, and capacity to respond to change, and ultimately result in better services and health outcomes. However, the programme theory is based on LDP content alone and does not account for differential impacts in various contexts.A review of the LDP suggests that it draws upon CQI principles in its approach. The LDP acknowledges the complex environment of managerial decision-making, and states that sustaining advances in health outcomes only occurs when leading and managing practices are absorbed into routine practise (i.e., their institutionalisation). However, the LDP is not explicit about this theoretical basis in CQI, nor does it claim systems thinking as a prime objective. We recognise that CQI philosophy – and implicitly, systems thinking – is embedded within the LDP practise and tools, and we were therefore interested in understanding the degree to which the LDP can stimulate systems thinking in district teams. Though the language differs, the concepts of systems thinking, CQI, and the LDP overlap in their approach to shifting problem-solving towards a more systemic orientation for improved decision-making: if systems thinking is the capacity to see interrelationships between components of a system, CQI is the process of managing these interrelationships, and the LDP is a practical intervention to implement these principles. This overlap is illustrated in Figure 1.

**Table 3:** LDP leading and managing practices

**Leading practices**	
Scanning	Identifying client priorities and needs
	Seeing opportunities, trends, constraints and risks
	(*Organisational outcome*: *valid*, *current knowledge of context*)
Focusing	Developing shared goals
	(*Organisational outcome*: *articulated mission*, *vision*, *strategies and priorities*)
Aligning/mobilising	Building congruence between values, mission, structures and daily actions
	Supporting teamwork
	(*Organisational outcome*: *external and internal stakeholders have ownership over organisational goals and support resource mobilisation towards these goals*)
Inspiring	Building trust and acknowledging team members
	Modelling creativity and learning
	(*Organisational outcome*: *climate of continuous learning with committed staff*)
**Managing practices**	
Planning	Identifying goals, annual plans and performance objectives
	(*Organisational outcome*: *defined results and matching resources*)
Organising	Ensuring accountability and authority structures
	Aligning staff capacities with planned activities
	(*Organisational outcome*: *functional structures and processes for operations*)
Implementing	Integrating workflows and systems
	Balancing competing demands
	(*Organisational outcome*: *effective*, *efficient and responsive actions*)
Monitoring and evaluation	Reflecting on progress against action plans
	Improving work processes and procedures
	(*Organisational outcome*: *continuous up*-*to*-*date data for decision*-*making*)

(*Adapted from Mansour* et al*.*, *2005*[19]).

The objectives of our study, therefore, are to understand: a) the mechanisms by which a complex intervention introduced into a complex context brings about its observed outcomes (i.e., how and why does the LDP ‘work’ when it is introduced into a district health system in Ghana); and b) whether or not the LDP increases systems thinking in district managers in this context.

## Methods

### Study setting

The LDP was introduced in a rural district, Dangme West^c^. The study took place from November 2011 to August 2013. An initial period of participant observation prior to the onset of the intervention lasted from November 2011 to January 2012. The LDP intervention was implemented from February to August 2012, and a follow-up period of participant observation extended until August 2013.

District managers were defined as: i) members of the district health management team (DHMT); ii) members of the district hospital management team; and iii) members of the three sub-district health teams. These managers were selected because they represent top-level management for decision-making within the district.

### Description of the LDP intervention in Dangme West

The LDP was introduced to district teams (district health administration, district hospital, and three sub-district management teams) by a facilitation team consisting of three members of the regional health administration, and one external consultant specialised in the LDP. The curriculum, teaching materials, and learning strategies were based on the LDP Handbook [19]. The LDP consists of a six-month cycle of root challenge identification, action planning, and monitoring and evaluation. For each training workshop, district teams consisted of 4 to 7 members per team, depending on the size of the facility. These were managers (core management including medical superintendents, district directors of health services, deputy directors of nursing services, physician assistants, and hospital administrators), and staff (accountants, public nursing officers, and midwives). Two-day, face-to-face workshops were held in the capital city Accra three times bi-monthly. These involved modules on LDP practices, developing a shared team vision, diagnosing challenge root causes, developing action plans, setting priorities, mobilising stakeholders to commit resources, monitoring and evaluation, understanding roles in teamwork, and building trust. Workshops were interspersed with monthly coaching visits, with the facilitation team attending teams and their wider staff in their facilities to ensure organisation-wide diffusion of LDP teachings. For their LDP results, each team identified one MNH-related challenge they wished to address (Table 4). Every team attained their planned results except for one sub-district team; at the time, the health facility did not have a resident midwife and thus faced difficulties in improving its skilled delivery coverage.

**Table 4:** **LDP results (short**-**term outcomes) February to August 2012**

**Team**	**LDP challenge**	**LDP results** (**short**-**term outcome**)
District Health Administration	Increase skilled delivery from 37% to 40%	Increased skilled delivery to 51%
District Hospital	Reduce still birth from (n=) 30 to 20	Reduced still birth to (n=) 11
Sub-district 1	Increase skilled delivery from 15% to 18%	Increased skilled delivery to 19%
Sub-district 2	Increase skilled delivery from 1.7% to 5%	Increased skilled delivery to 2.6%
Sub-district 3	Increase focused antenatal care from 0 to 20%	Increased focused antenatal care to 22%

### Study design: realist evaluation

We used a case study design as most appropriate for organisational studies in which ‘how’ or ‘why’ questions are being asked. Criticisms of case study designs include their weak external validity [25]. Seeking to address this criticism through cumulative validation, realist evaluation is an approach capable of addressing complex investigation and probing causal linkages between contexts, actors, and the changes observed. Realist evaluation attempts to move beyond asking ‘did the intervention work?’ towards understanding ‘how did the intervention work, for whom, and in which contexts’ [26-29]? The case study begins with the formulation of the middle range theory (MRT), based on existing theory and past actor experience. The MRT, structured as a ‘context + mechanism → outcome’ (CMO) configuration, is validated with actors, and against the literature. The validated MRT then becomes the working hypothesis to be ‘tested’ in the case. It is subject to revision based on accumulated new evidence.

### Analytical framework: our middle range theory

#### Context of district manager decision-making (C)

The first part of developing our MRT included an in-depth exploration of the decision-making context for district managers in Ghana. Based on our pre-LDP observation period, we found that district managers have narrow decision-space due to the highly-centralised authority within the GHS. National-level control over resources leads to resource uncertainty at district level. Through formal and informal communication channels, district managers engender trust and employ it as a coping mechanism to counter organisational uncertainty and manage the risk of not fulfilling their managerial mandates of oversight, coordination, and networking in the face of resource scarcity. Trust and respect for regional- and national-level authorities further legitimises the system’s hierarchy, thereby reinforcing it [unpublished observations]. This decision-making ‘loop’ is the context into which the LDP was introduced.

#### Outcomes of the LDP – short- and medium-term (O)

Furthermore, we worked backwards from the observed short-term outcomes of the LDP (i.e., LDP results) and medium-term outcomes, which were interpreted as the residual organisational changes (i.e., LDP institutionalisation). These included new organisational roles and relationships as a result of the LDP, extensiveness (i.e., how widely disseminated across the organisation) and intensiveness (i.e., how deeply integrated into routine practise) of the LDP, and any organisational routines displaced by the LDP [30].

#### Mechanisms of the LDP (M)

Through our MRT, we attempt to uncover the mechanisms of the LDP. Our beginning assumption was that if systems thinking took place as a result of LDP practices, this would support LDP institutionalisation. In a feedback mechanism, institutionalisation of the LDP would further increase systems thinking. We hypothesised our MRT as follows:

The LDP brings about its short-term outcomes by encouraging district managers to seek alternative sources of financial and material resources. If successful, the increased ability to look within and across the district for resources: i) supports relationship building with district stakeholders, which improves the number and quality of district relationships; ii) expands managerial understanding of the linkages and interactions in the district health system, which deepens systems thinking in managers, and supports LDP institutionalisation; and iii) reduces resource uncertainty, which lessens managerial risk, and thus the need to draw upon trust and respect as coping mechanisms. Reduced resource uncertainty increases district manager decision-space. Reduced uncertainty triggers the LDP’s underlying focus on organisational control.

### Rival MRT

We also propose a rival MRT where the LDP brings about its short-term outcomes by reinforcing hierarchical authority, because it is introduced in a top-down manner. As such, resource uncertainty remains high, and district manager decision-space narrow. Thus, district managers continue to rely on trust and respect as coping mechanisms to deal with resource uncertainty. The context of high uncertainty triggers the LDP’s underlying focus on organisational creativity. This focus on creativity stimulates systems thinking, which supports LDP institutionalisation.

### Data collection

#### Document review

For data on the LDP implementation, we reviewed weekly district management team meeting minutes and monthly regional management team meetings for the duration of the study period, as well as all training workshop materials, team presentations and action plans, and reports from previous LDP cycles in other regions. For overall context, we further reviewed national, regional and district policies, and protocols (Additional file 1: Table S1).

#### Participant observation

For the duration of the study period, the first author participated in weekly district health management meetings, monthly regional health management team meetings, semi-annual district planning and district review meetings, all LDP training workshops and coaching visits, teams’ LDP activities, DHMT supervisory visits to sub-districts, and day-to-day operations of the district. Until October 2012, the third author participated in monthly regional health management team meetings. Continuous discussion with management and staff was the method of sense-making used. As part of their routine management meetings, validation workshops took place at the end of the initial and follow-up observation periods to feedback findings to district teams and integrate their views into the analysis.

#### Semi-structured interviews

We conducted a total of 23 interviews with members of the DHMT (8), district hospital management (4), and sub-district management (7); 4 managers were lost to staff transfers (2 from the DHMT and 2 at the sub-district level). At the regional level, we interviewed 3 out of 4 members of the LDP facilitation team, and one development partner supporting the LDP; 17 respondents were women and 6 were men; 3 respondents were in their current posting less than 1 year, 13 between 1–3 years, and 7 between 3–5 years. More than half the respondents (12) had no prior formalised management training.

Interview guides were developed to investigate team perceptions of quality, actual LDP implementation (including challenges and functioning), influence of concurrent district initiatives, organisational sustainment of LDP practices, and changes in relationships and resources. Interviews took place 8 months after the end of the LDP.

### Data analysis

Audio-recorded interviews were conducted in English, and observational field notes were converted into transcripts, cleaned, and entered into Atlas.ti^©^ qualitative analysis software. Transcripts were coded against an initial start-code list developed from systems thinking, LDP, CQI concepts, and our MRTs. Emerging themes from the data were also coded. In order to ‘configure’ our CMOs [31], we began with the short-term outcomes. We triangulated across data type and source to systematically arrange our medium-term outcomes and unearth potential mechanisms of the LDP. We then drew out linkages between the contexts, outcomes, and identified mechanisms in a causal loop diagram (CLD).

### Ethical considerations

This study was part of a larger study to identify effective ways of improving MNH service delivery, for which ethical approval was awarded by the Ghana Health Service Ethical Review Committee. Teams were made aware of the observation periods. Respondents participated voluntarily, and were able to withdraw at any time. Informed consent was obtained from all respondents, and respondent anonymity was maintained during all parts of the study using coding.

## Results

### LDP as it was implemented

The LDP was mainly implemented as designed. During implementation, the LDP was frequently discussed as part of management team meetings, and was often mentioned at the monthly regional health management team meetings.

The LDP training approach was more team-based, less didactic, and more intensive than most district workshops. Modules focused more on the deployment of LDP tools and proceeding through LDP processes, and less on facilitating teams to reflect on their own organisational practices or thinking systemically through them. This was indicated in the first LDP workshop, where facilitators identified the programme goals as being: i) to learn how to lead and manage to enable others to face challenges and achieve results; ii) to apply tools to analyse challenges to achieve results; iii) to know how to produce measurable results; and iv) as managers, to learn how to build a positive work climate. The emphasis was more on the LDP’s ‘managing’ rather than ‘leading’ practices.

A review of teams’ LDP action plans and presentations showed that teams broadly undertook two categories of activities: i) community sensitisation and customer care training for frontline staff, or ii) lobbying local organisations for material resources. From the customer care training workshops we found clear patterns of hierarchy being reinforced. Customer care workshops were facilitated by non-LDP regional staff and were regarded as ‘customer care as corporate responsibility, to redeem the corporate image’. In part, this stemmed from some high-profile media cases about staff error. Emphasis was placed on rules and regulations of the GHS, proper comportment of staff in forms of address towards their seniors, and dress codes. Very little related to client-provider relationships and there was minimal opportunity for staff to reflect on their experiences with clients. Furthermore, in performing their root cause analyses, teams were not trained to investigate the interrelationships between different causes, but rather to deal with single root causes separately. Taking the example of poor staff attitude, teams worked through their root cause analysis in the following manner:

“*staff attitude is poor*, *because staff lack courtesy and good customer care*; *this is because they have inadequate knowledge about good customer care*; *which is because they have not been trained on good customer care*; *therefore the solution is to provide customer care training*.”

An example of gaps between LDP practise in the context of its implementation and LDP practise in routine work was observed 2 weeks prior to the final LDP workshop. In one sub-district, having been called to assist in a conflict between staff and management, DHMT members resolved the situation by stating:

“*Any time your leader tells you something*, *she has a plan. Only one person can lead*, *others follow faithfully. Yours is to do what you are told. The rest*, *she will manage*”.

Once ended, there was little evidence of teams’ efforts to support LDP institutionalisation. None of the five teams engaged in another LDP cycle, no new staff were oriented in the LDP, no funds were set aside for LDP activities, and meeting minutes and staff conversations no longer reflected mention of the LDP. The lack of team efforts towards LDP institutionalisation was influenced to some extent by time constraints of routine district work: at the time the LDP ended (August–September), district teams were focused on completing year-end activities and reporting, and preparing for a new planning cycle. LDP institutionalisation was further compromised by changes of leadership at regional, district, and sub-district levels, which witnessed the appointment of new directors at each level. Critically, the splitting of the district into two separate districts in October 2012 required new administrative structures in the new district, and a restructuring of relationships across both districts. It does not appear that teams used their LDP practices to support these transitions. Several months after the end of the LDP, the majority of team members could not list the LDP practices. The LDP did not appear to support the development of systems thinking in district managers.

### Participant perceptions of the LDP

The introduction of the LDP from the region was unexpected by district teams, and was not initially part of their annual work plan. However, in the context of verticalised programming, this is common. The facilitation of the LDP by the region was perceived in two distinct ways. From the regional perspective, facilitating the LDP provided an opportunity to remind district teams of ‘proper conduct’, part of which was complying with regional directives. From the district perspective, having regional facilitators participate during coaching visits, heightened the experience:

“*These big*, *big*, *top*, *top*, *top*, *people were here. It*’*s not the normal people like we that they* [the staff] *are used to. So that one alone will give them some inspiration*…” (DHMT member)

Since the teams had little formalised management training, the novelty of the LDP disposed them to being receptive to capacity support. The exposure to management practices enabled teams to attain their LDP results, and they noted that the imposition of deadlines created a sense of urgency and increased the need to attain results, compared to their routine targets. The LDP also helped managers build initiative. Managers acknowledged that some problems were ‘beyond’ them, and therefore, initiative-taking was encouraged, but only on a ‘small-scale’:

“*You are supposed to make do with what you have. Because sometimes when we have challenges we think that* ‘*oh as for this one*, *we are waiting for region to come and do it*, *or we are waiting for national to come and do it*’. *LDP says you shouldn*’*t think so big*, *but something within*… *you should just try to think around yourself*”. (DHMT member)

Managers learned to better prioritise and felt more able to manage concurrent programmes, and thus viewed themselves as working more efficiently. Supporting teamwork through inspiration and acknowledgment was also important. One manager stated that prior to the LDP she used to ignore her staff if they incorrectly performed a task. Managers did note that the LDP had no influence on the relationship between district and regional levels, nor did it alter the dynamics around resources:

“*It hasn*’*t changed our resources. If I am saying the truth*, *I don*’*t think we have the resources to work with*”. (Sub*-*district head)

One issue reported consistently by district managers was the LDP’s resource intensiveness. Convening stakeholders and running training workshops all require additional funds, which was perceived as burdensome, since teams had severe resource constraints and had not budgeted for the LDP in advance. Lobbying for funds from the District Assembly was difficult as the annual planning cycle had already passed. Furthermore, the time required to meet for LDP activities, convening the wider team, and preparing plans was viewed as onerous in the face of concurrent programmes and other health system constraints. One manager highlighted the difficulty that under-staffing created in trying to gather staff for training without disrupting service delivery:

“*The challenges that we had in implementing the LDP were trying to get staff themselves to come around to listen to us. It*’*s terrible*, *the beginning it was very hard to get the unit heads to come around. The reason was that due to lack of staff. The unit heads must be there to monitor*, *and there is no staff to bring to come and listen to us*.” (Hospital management)

Managers widely perceived the lack of LDP institutionalisation as related to the LDP being a ‘regional project’:

“*You can also see that at the regional level it has ended. So if the regional level it has ended can the district continue*? *Since then there has never been any coach from region to come and see what we have done*, *where we have reached and what the challenges are. So you can imagine*, *we at the sub*-*district can we also do it*? *So me*, *it is not about the district not doing it or it*’*s not implementing it*, *I only see it as a project*…*at the* [LDP workshops] *we were told it is not a project*, *it is a running thing. But it has ended as if it is a project and the project has come to an end*.” (DHMT member)

### Proposing causal linkages (C + M → O)

**Figure 2:**
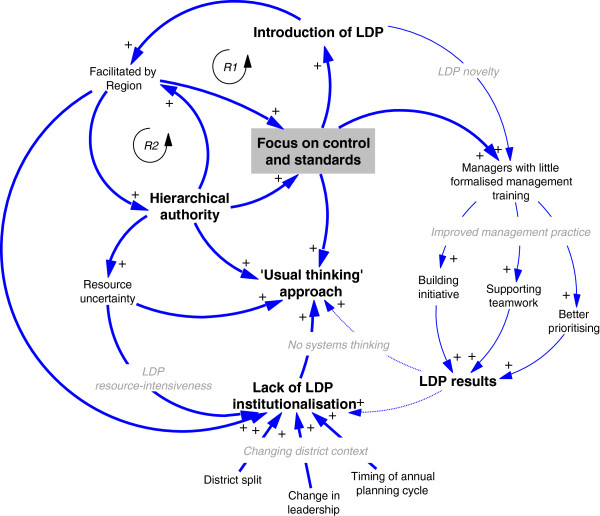
**Causal loop diagram of LDP implementation**, **February to August 2012.**

We illustrate the relationships between our contexts, mechanisms, and outcomes in a CLD^d^ (Figure 2). This schema represents causation between variables, with directions of influence depicted by arrows. Influence in the same direction is represented by positive arrows. Feedback loops can reinforce (R) or self-regulate the pathway [32].

**Figure 3:**
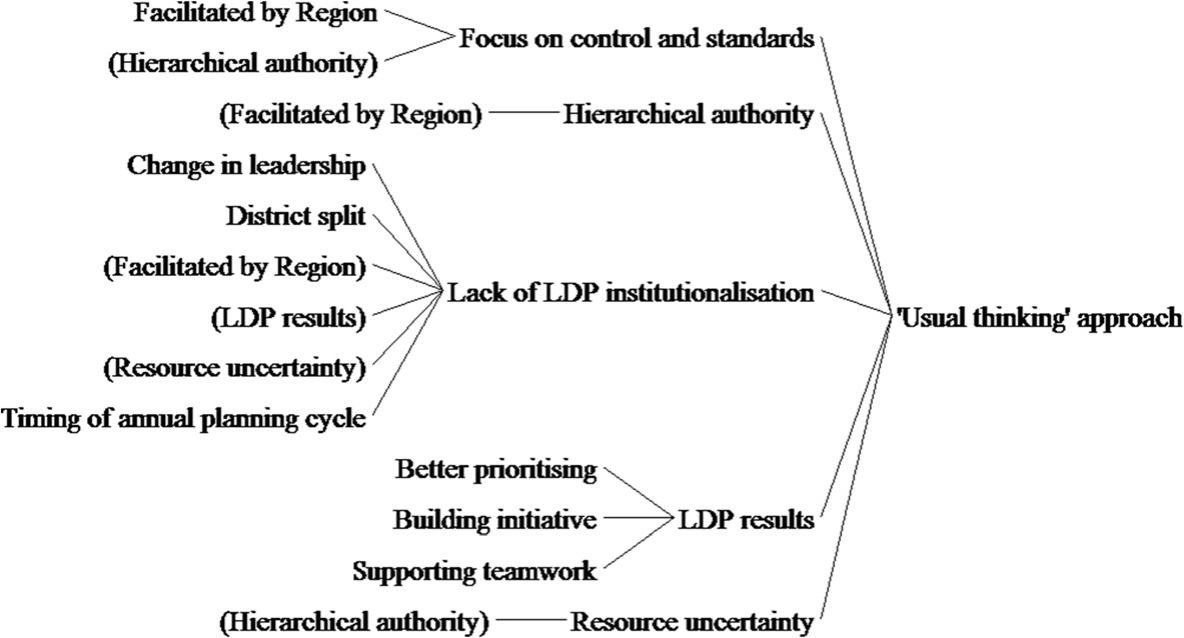
**Causal tree diagram of LDP implementation**, **February to August 2012.**

Our CLD shows the pathway of the LDP’s short-term outcomes (right-side of the figure, thin arrows) and medium-term outcomes (left-side of the figure, thick arrows). On the short-term, the novelty of the LDP for managers with limited formalised management training (C) stimulated the value and utility of bundled management practices taught by the LDP (M) for teams to achieve their LDP results (O). This causal pathway is linear, and does not significantly deviate from the predicted programme theory of the LDP. On medium-term outcomes, the introduction and facilitation of the LDP in a top-down manner (i.e., from the region) (C) promoted hierarchical authority and triggered the LDP’s focus on controls and standardisation (M). Multiple, reinforcing feedback mechanisms (R1 and R2) neither supported LDP institutionalisation, nor systems thinking among district teams (O).Had our original assumption been borne out, we would see a third reinforcing loop (R3) between systems thinking and LDP institutionalisation. For simplicity sake, we redraw the same analysis as a causal tree diagram (Figure 3).

## Discussion

Hawe et al. [30] suggest that the most important dimension of complexity is frequently not the complex intervention itself, but rather the context into which it is introduced. We found that in trying to produce change in a complex adaptive system, the LDP in this case could not be sufficiently institutionalised. In essence, the system ‘rejected’ it and returned to its prior equilibrium. The context of system hierarchy, as demonstrated by the deployment of regional staff to train the districts, highlights the cascading approach to systems change from the top. This may not always be appropriate, and further underscores the need to think systemically when introducing any intervention. We note that, in this case, the LDP appeared to engage systems thinking in its tools rather than through its practices, and incorporated its CQI elements in its organisational outcomes rather than its processes. This suggests a focus on organisational control, rather than creativity, of both the LDP and the organisational context into which it was introduced. Being tool-driven, the LDP does not itself provide processes for developing a learning organisation, and we noted no evidence of new mental models created in district teams – what Sterman [33] distinguishes as ‘single-loop’ versus ‘double-loop’ learning. Our study raises questions about the nature of management and leadership capacity strengthening. We recognise that short-term capacity strengthening interventions may not necessarily support such reorientations. As such, it is critical for donor partners and national governments to reconsider the types of idealised interventions often put in place, and how contexts can modulate expected outcomes over time. This suggests support for longer term, more reflective, and potentially unpredictable capacity strengthening approaches. This notion is further supported by a recent study from Rwanda that found no statistical association between training and adherence to recommended MNH practise [34]. Our findings uphold earlier work by Blaise and Kegels [35], who describe the rigidity and lack of responsiveness in command-and-control structures observed in several African health systems as contributors to quality of care challenges in service delivery.

At the outset we hypothesised that, in reinforcing system hierarchy, the LDP’s underlying goal of organisational creativity would prevail due to the context of high uncertainty. Paradoxically, the LDP’s underlying goal of organisational control was more pronounced. We attribute this to the degree of centralised decision-making in the system: the strength of ‘command-and-control’ overrides other mechanisms that enable learning, creativity, and adaptability. This mismatch in contextual uncertainty and organisational culture may very well account for the lack of effective management at district level. With this in mind, we refine our MRT as follows:

The LDP brings about its short-term outcomes through its experience of novelty, building initiative, supporting better prioritisation, and building teamwork. The LDP reinforces hierarchical authority due to being introduced in a top-down manner. As such, resource uncertainty remains high and, as a consequence, district manager decision-space remains narrow. Thus, district managers continue to rely on trust and respect as coping mechanisms to deal with resource uncertainty and their managerial risk. The context of high uncertainty, coupled with reinforced hierarchical authority, triggers the LDP’s underlying focus on organisational control. Systems thinking is not stimulated, and LDP institutionalisation does not occur.

In thinking about how the LDP might have been implemented differently, we consider five ways in which the causal pathway could have been altered: i) had the LDP facilitators been peers instead of superiors (for example, training teams could have consisted of district managers whose districts had previously undertaken the LDP rather than being regional officers), this may have weakened hierarchical authority, thereby reducing the top-down nature of its introduction; ii) had districts volunteered to receive the LDP instead of being randomly selected, they may have expected it and better prepared their resources; iii) had ongoing mentorship and coaching been built into the process through systematic follow-up, this may have supported the view of greater district ownership; iv) had the timeframe of the LDP intervention been lengthened to include two or three cycles, this may have had longer-lasting effects and become routine practise; and v) had organisational creativity and learning been an explicit goal, with reflective processes as a major part of the intervention, this may have provided greater opportunity for more systems thinking to develop in district managers. We recommend that the LDP could be strengthened by a more explicit integration of CQI philosophy and principles into its existing tools, and greater attention paid to context to support its institutionalisation. We are aware of existing CQI-based interventions in the Ghanaian health system with similar ‘Plan-Do-Study-Act’ cycles, indicating that the lack of institutionalisation of one programme does not prevent the implementation of other similar interventions.

Our findings clearly demonstrate that a lack of consideration of the context into which such interventions are introduced can minimise their effectiveness. More importantly, our work highlights the fact that context also informs the kind of management and leadership that emerges at district level. Not uniquely a Ghanaian challenge, decision processes are often rooted in a desire for control and prediction, such that managers who cannot deliver are perceived as ineffective and are soon replaced [36]. These issues exceed the scope of our study, but do underscore the fact that improvements in management and leadership do not reside in the capacities of managers alone, but demand keen attention to the organisational contexts in which managers are embedded.

A limitation of our study is that it reports on only one context for LDP implementation. This is a first level analysis; moving forward we expect to conduct a wider exploration of other districts in the Greater Accra Region and further refine our MRT.

## Conclusions

The influence of contexts on mechanisms in the gap between short- and medium-term outcomes is particularly important given that decisions to scale-up interventions are frequently based on their success in the short term. In the Ghanaian context, introducing the LDP into a context of highly centralised decision-making and resource uncertainty triggered its underlying goal of organisational control. More explicit focus on systems thinking principles that enable district managers to better cope with their contexts may strengthen the institutionalisation of the LDP in the future.

## Endnotes

^a^The Ghana Health Service (GHS) is the semi-autonomous agency of the Ministry of Health responsible for public health service delivery. Since its creation in 1996, the GHS has been administratively decentralised along national, regional, and district lines. National-level GHS is responsible for policy direction. Regional health directorates provide technical and administrative oversight to district health directorates responsible for coordinating service provision at district and sub-district level.

^b^This was as part of a larger study to improve service delivery for MNH. One key aspect of the study sought to support managerial decision-making as part of the overall organisational change needed to improve MNH quality in the Greater Accra Region. The larger study envisioned designing and implementing a CQI programme to address this. However, given the existence of the LDP already in the health system, and its overlap in approaches and philosophy with CQI, the LDP was instead identified as the CQI-based intervention to be evaluated.

^c^Dangme West was subsequently divided into two separate districts: Shai-Osudoku District and Ningo-Prampram District; this paper reports its findings against the district structure at the time of the study.

^d^We drew Figures 2 and 3 using Vensim simulation software : http://www.vensim.com.

## Abbreviations

CQI: Continuous quality improvements; CLD: Causal loop diagram; CMO: Context-mechanism-outcome configuration; DHMT: District health management team; GHS: Ghana Health Service; LDP: Leadership Development Programme; LMIC: Low- and middle-income country; MNH: Maternal and newborn health; MRT: Middle range theory.

## Competing interests

The authors declare that they have no competing interests.

## Authors’ contributions

Conceived and designed study: AK, IAA, HvD. Developed instruments and collected data: AK, IAA. Analysed data: AK, IAA, HvD. Wrote and reviewed manuscript: AK, IAA, HvD. All authors read and approved the final manuscript.

## Supplementary Material

Additional file 1: Table S1 Policy documents and reports reviewed. Click here for file
