# Supplementary material for: Advancing the application of systems thinking in health: realist evaluation of the Leadership Development Programme for district manager decision-making in Ghana
Source: Health Res Policy Syst. 2014 Jun 16;12:29. doi: 10.1186/1478-4505-12-29 (PMC4073809; doi:10.1186/1478-4505-12-29)
Supplement: Additional file 1: Table S1 — Policy documents and reports reviewed. [file 1478-4505-12-29-S1.pdf]

Additional files

Additional File 1. Policy documents and reports reviewed

Document Name	Document Type, Year
National Health policy	National policy document, 2007
Human Resource Policies and Strategies for the Health Sector (2007-2011)	National policy document, 2007
Ghana Health Service Reproductive Health Strategic Plan (2007-2011)	National policy document, 2007
Health Sector Gender Policy	National policy document, 2009
Ghana MDG Acceleration Framework and Country Action Plan	National policy document, 2011
Ghana Health Sector Programme of Work (2012-2014)	National policy document, 2011
Maternal Health/Death Audit Guidelines	National protocols/guidelines, 2002
Safe Motherhood Service Protocols	National protocols/guidelines, 2008
Referral Policies and Guidelines	National protocols/guidelines, 2010 (Reprint)
National Consultative Meeting on the Reduction of Maternal Mortality in Ghana: Partnership for Action	National report, 2008
Ghana Maternal Health Survey	National report, 2009
Ghana Millennium Development Goals Report	National report, 2010
Ministry of Health Annual Report	National report, 2010
Independent Review Health Sector Programme of Work (2009)	National report, 2010
Independent Review Health Sector Programme of Work (2010)	National report, 2011
National Assessment for Emergency Obstetric and Newborn Care	National report, 2011
Health of the Greater Accra Regional	Regional report, 2011
Dangme West District Annual Report and Review Health Sector Performance	District report, 2010
Dangme West District Annual Report and Review Health Sector Performance	District report, 2011
Dangme West District Programme of Work (2012-2014)	District policy document, 2011
Regional Health Management Team Meeting Minutes	Regional minutes, 2011-2012
District Health Management Team Meeting Minutes	District minutes, 2011-2012
